# DNA Methylation and Chromatin Regulation during Fleshy Fruit Development and Ripening

**DOI:** 10.3389/fpls.2016.00807

**Published:** 2016-06-14

**Authors:** Philippe Gallusci, Charlie Hodgman, Emeline Teyssier, Graham B. Seymour

**Affiliations:** ^1^EGFV, Bordeaux Sciences Agro, INRA, Université de Bordeaux Villenave d’Ornon, France; ^2^School of Biosciences, University of Nottingham Sutton Bonington, UK

**Keywords:** DNA methylation, epigenetics, ripening, tomato, crop improvement

## Abstract

Fruit ripening is a developmental process that results in the leaf-like carpel organ of the flower becoming a mature ovary primed for dispersal of the seeds. Ripening in fleshy fruits involves a profound metabolic phase change that is under strict hormonal and genetic control. This work reviews recent developments in our understanding of the epigenetic regulation of fruit ripening. We start by describing the current state of the art about processes involved in histone post-translational modifications and the remodeling of chromatin structure and their impact on fruit development and ripening. However, the focus of the review is the consequences of changes in DNA methylation levels on the expression of ripening-related genes. This includes those changes that result in heritable phenotypic variation in the absence of DNA sequence alterations, and the mechanisms for their initiation and maintenance. The majority of the studies described in the literature involve work on tomato, but evidence is emerging that ripening in other fruit species may also be under epigenetic control. We discuss how epigenetic differences may provide new targets for breeding and crop improvement.

## Introduction

The fruit is an organ that is unique to the Angiosperms or flowering plants and a true fruit is defined as a mature ovary, although accessory tissues can form the bulk of the fleshy fruit tissue in some cases (Seymour et al., 2013). Ripening in fleshy fruits involves a profound phase change in the leaf-like tissues that encase or are associated with the mature seeds and it can completely alter the metabolic state of a carpel organ or associated tissues. Recent discoveries indicate that ripening is under both strict genetic and epigenetic control.

Epigenetics refers to heritable changes in gene expression that occur without modification of the underlying DNA sequence. It involves histone Post-Translational Modifications (PTMs) and DNA methylation which are transmitted through DNA replication and cell propagation, thereby determining and maintaining cell-type specific gene expression patterns (Vermaak et al., 2003; Chan et al., 2005; Reyes, 2006; Li et al., 2007; Eichten et al., 2014; Pikaard and Mittelsten Scheid, 2014). We do not discuss alterations in small RNA composition or abundance in any detail because the relationship between inherited small RNA levels and fruit development and ripening has been little studied and their general role in plant development has been the subject of recent reviews (for example Borges and Martienssen, 2015). Studies in *Arabidopsis* and other plants, including tomato have demonstrated the relevance of epigenetic mechanisms in the control of plant developmental processes (Choi et al., 2002; Hsieh and Fischer, 2005; Lauria and Rossi, 2011) and their potential impact on traits of agronomical interest such as flowering time (for a review He G. et al., 2011), heterosis (Dapp et al., 2015), and fleshy fruit ripening (Manning et al., 2006; Zhong et al., 2013; Liu et al., 2015). So far, much of the work analyzing the impact of epigenetic regulation on fleshy fruit quality has been undertaken mainly in tomato (*Solanum lycopersicum*), because this is the model system for investigating the molecular basis of ripening in fleshy fruits. Even in this fruit the extent and role of the epigenetic regulation of ripening is still relatively poorly understood. Here, we review the available literature and identify areas for further investigation. The limited information on the potential role of histone PTMs in fruit development and ripening is discussed, but the review focuses on recent evidence demonstrating that DNA methylation plays a crucial role in ripening. Major questions that need to be addressed include the nature, extent and stability of epigenetic variation that may impact ripening and whether epigenetic control of this process is a common feature of all fruit bearing species. A better understanding of epigenetic control of ripening has the potential to provide novel strategies for generating sources of variation for crop improvement.

## Histone Post-Translational Modifications May Have Important Functions in Fleshy Fruits

Post-translational modifications of histones influence chromatin organization and contribute to the epigenetic regulation of gene expression. Histone PTMs include phosphorylation, methylation, acetylation, or ubiquitination and depend on a wide range of enzymes that determine their genome wide distribution and abundance (reviewed in Berr et al., 2011). So far, four major chromatin states, corresponding to specific combinations of 11 different histone PTMs and of DNA methylation, have been determined in *Arabidopsis* that are preferentially associated with active or repressed genes, intergenic regions and transposons (Roudier et al., 2011). These chromatin states appear similar to the situation described in *Drosophila*, although five different chromatin states were defined in this case (Filion et al., 2010). In addition, some marks seem preferentially associated to specific chromatin states. For example, histone acetylation is preferentially linked to gene expression whereas dimethylation at lysine 9 of histone H3 seems to correlate with constitutive heterochromatin and trimethylation of lysine 27 with gene repression (Roudier et al., 2011). There are many enzymes that participate in PTMs and the functions of a few of them are starting to be deciphered, mainly in the model plant *Arabidopsis* (For a review, Berr et al., 2011). In this case, it is becoming clear that histone PTMs are critically important for several aspects of plant development and adaptation to stress (for reviews see Ahmad et al., 2010; Mirouze and Paszkowski, 2011; Eichten et al., 2014), but no direct effect on *Arabidopsis* fruit development has been documented so far.

**FIGURE 1:**
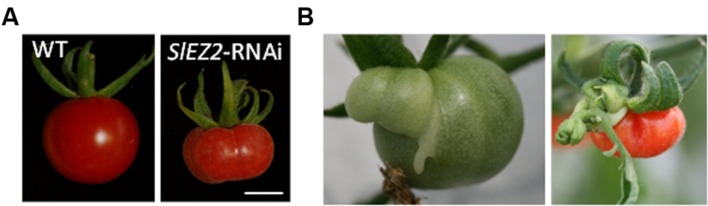
**(A)** Red ripe *SlEZ2*-RNAi tomato fruits are characterized by modified shape, colour, and surface aspect (right) compared to the WT fruits (left; bar = 1 cm). **(B)** *SlEZ2*-RNAi plants occasionally develop fruits with extra carpels. In some cases ectopic flowers and/or leaves (left) are also observed. **(A)** Courtesy from P Gallusci and E Teyssier **(B)** Adapted from Boureau et al. (2016).

Several recent studies have described the expression pattern of histone modifiers, including histone deacetylases (HDACs), histone acetyltransferase (HATs), or histone methyl transferases (HMT) in a range of fleshy fruits including apple (Janssen et al., 2008), citrus (Xu et al., 2015a), grape (Aquea et al., 2010, 2011; Almada et al., 2011), and tomato (Cigliano et al., 2013; Zhao et al., 2014). The results indicate that some of the genes involved in histone PTMs are preferentially or specifically expressed in fruits and may present stage preferential expression, suggesting their recruitment for the regulation of fruit development. For example, a few tomato *HMT* genes, among which those encoding the ENHANCER OF ZESTE [E(z)] proteins, were shown to be expressed during early phases of tomato fruit development (How Kit et al., 2010; Cigliano et al., 2013) suggesting an early programming of chromatin structure necessary for proper fruit development. This is consistent with the functional analysis of the two tomato *SlEZ1* and S*lEZ2* genes which encode the tomato E(z) proteins orthologous to the *Arabidopsis* SWINGER and CURLY LEAF, respectively (How Kit et al., 2010; Boureau et al., 2016). E(z) proteins, together with EXTRA SEX COMB protein, FERTILISATION INDEPENDENT ENDOSPERM DEVELOPMENT (FIE) and the SUPPRESOR OF ZESTE 12; FERTILISATION INDEPENDENT SEED DEVELOPMENT 2 (FIS2) are the core elements of the POLYCOMB REPRESSIVE COMPLEXES 2 (PRC2s, **Table 1**), that govern transition phases during the development of *Arabidopsis* plants and determine cell type specificity (for a recent review: Mozgova and Hennig, 2015). Knock down of *SlEZ1* had no impact on tomato plant and fruit development, and resulted in alteration of flower shape and development of fruits with a moderate increase in carpel number suggesting that SlEZ1 is mainly involved in flower formation (How Kit et al., 2010). In contrast, *SlEZ2* repression led to fruits with modified shapes, texture and color, eventually presenting ectopic carpels (**Figure 1**; Boureau et al., 2016). Color alteration was due to reduced cutin content rather than to changes in carotenoid composition, and these cutin changes also resulted in a rapid shrinking of fruits when left overripe on plants. In addition, ripe *SlEZ2* RNAi fruits were characterized by a high trichome density as compared to WT fruits of the same age consistent with SlEZ2 being involved in the control of tomato fruit epidermal cell identity. It is noteworthy that, fruit shape, aspects of texture and cutin content are dependent on events occurring early during fruit development (Chaïb et al., 2007; Mintz-Oron et al., 2008; van der Knaap et al., 2014) and these events occur contemporaneously with the highest expression level of *SlEZ2* (How Kit et al., 2010; Boureau et al., 2016). These results indicate a more prominent role of the SlEZ2 protein in the control of fruit development and are consistent with polycombs being primarily involved in early stages of fruit development (Boureau et al., 2016). Interestingly, repression of the gene encoding the tomato FIE protein had a stronger effect than either of the *SlEZ* RNAi lines described above and resulted in parthenocarpic fruit development, modified flower and fruit shapes. As FIE is encoded by a unique gene in the tomato genome (Liu et al., 2012; Boureau et al., 2016), this protein is likely to participate in all PRC2 complexes; which may result in effects stronger than those caused by knocking down single *EZ* genes.

**Table 1:** Tomato genes encoding the proteins of the Polycomb Repressive Complex 2.

Gene accession (Solgene)	Gene id (NCBI)	Proposed names: actual review	Proposed *Arabidopsis* ortholog (gene id)	Reference
Solyc01g079390	100134891	SlEZ1	AtSWN (828165)	How Kit et al., 2010
Solyc03g044380	100134892	SlEZ2	AtCLF (816870)	How Kit et al., 2010; Boureau et al., 2016
Solyc02g093190/Solyc02g093200	101267964	SlEZ3	AtCLF (816870)	How Kit et al., 2010; Boureau et al., 2016
Solyc03g093640	100134887	SlEMF2	AtEMF2 (835198)	
Solyc07g064090	100136877	SlFIE	AtFIE (821622)	


Other evidence of chromatin regulation during fruit development and ripening comes from the study of the high pigment mutants in tomato, *hp1* and *hp2*. These are caused by lesions in the genes encoding the UV-damaged DNA binding protein 1 (DDB1) and de-etiolated-1 protein (DET1), respectively, and result in enhanced fruit color and levels of carotenoids in the pericarp (Mustilli et al., 1999; Liu et al., 2004). Both the *DDB1* and *DET1* gene products associate with Cullin 4 (CUL4) to form the CUL4-DDB1-DET1 complex (Chen et al., 2006), which plays a central role in controlling protein degradation. Evidence indicates that DET1 also binds to non-acetylated amino-terminal tails of the core histone H2B in the context of the nucleosome and is likely to be involved in transcriptional repression (Benvenuto et al., 2002; Fisher and Franklin, 2011). Interestingly, a methyl CpG binding domain protein (SlMBD5) was recently shown to physically interact with DDB1 in tomato. Overexpression of *SlMBD5* in tomato plants led to a fruit phenotype similar to the *hp1* loss of function mutant indicating that this protein and DDB1 have antagonistic effects in fruits. DDB1 together with DET1 and CUL4 inhibits gene expression whereas SlMBD5, following it’s binding to methylated CG, would act as a transcriptional activator (Li et al., 2015). Although the precise mechanisms and targets of the CUL4-DDB1-DET1 complex and SlMBD5 have not been identified yet, these results suggest a complex interplay between histone marks and DNA methylation in the regulation of fruit development and ripening (Li et al., 2015). Indeed, there is also strong evidence that DNA methylation *per se* plays an important role in the control of fruit development and ripening, as discussed below.

## DNA Methylation in Plants: An Overview

Epigenetic modifications involving changes in DNA methylation are the main focus of this review, because these types of changes have been demonstrated to be major regulators of fruit ripening. In eukaryotes, DNA methylation refers to the addition of a methyl group to the carbon 5 of cytosine [5-Methylcytosine (5mC)]. Changes in DNA methylation are associated with a wide range of biological processes such as gene and transposon silencing (Law and Jacobsen, 2010; He G. et al., 2011; He X.-J. et al., 2011). These also include the control of maternal imprinting (FitzGerald et al., 2008; García-Aguilar and Gillmor, 2015) and homologous recombination during meiosis (Mirouze et al., 2012; Yelina et al., 2015). Indeed, plants with experimentally induced hypomethylated genomes present several developmental defects (Finnegan et al., 1996) consistent with DNA methylation being essential for proper plant growth. It is only recently, however, that an understanding of the central role for DNA methylation in controlling traits of agronomical relevance has begun to emerge, among which its role in responses to biotic and abiotic stresses (Baulcombe and Dean, 2014; Probst and Scheid, 2015), heterosis (Shen et al., 2012), and ripening in tomato and other fleshy fruits (Manning et al., 2006; Teyssier et al., 2008; Msogoya et al., 2011; Zhong et al., 2013; Liu et al., 2015; Xu et al., 2015b) are important examples.

**FIGURE 2:**
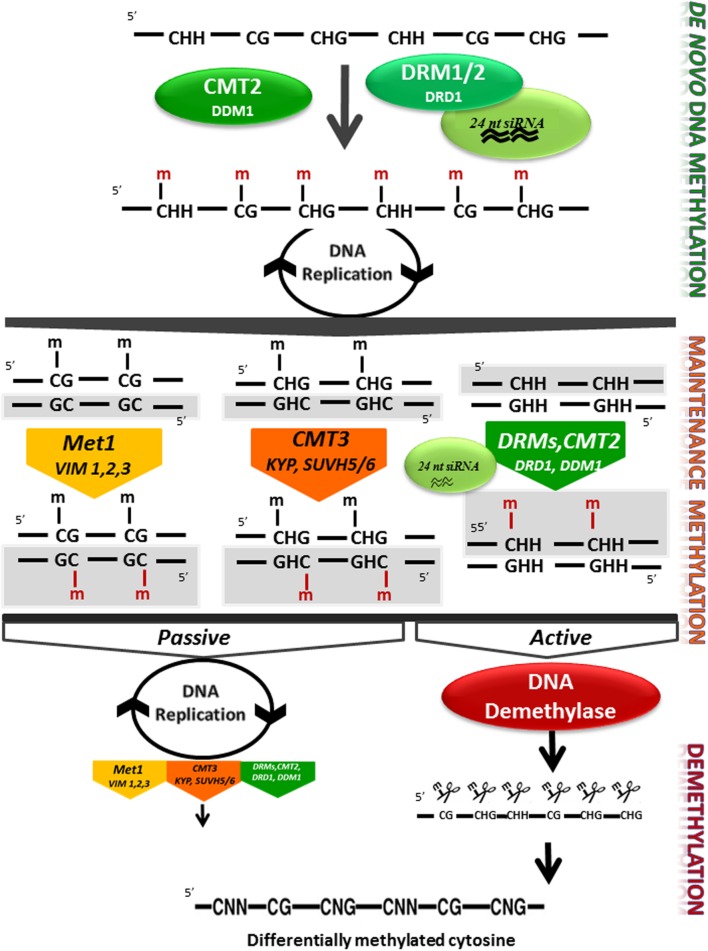
**DNA methylation control in plants.** Methyltransferases and DNA demethylases are involved in 5mC *de novo* methylation, maintenance methylation, and demethylation in higher plants. *De novo* DNA methylation is set up by the RNA directed DNA Methylation (RdDM) pathway involving the DRM1/2 methyltransferases, DRD1 and 24 nt long small RNAs, and by the chromomethylase CMT2 with DDM1 in the CHH sequence context at heterochromatic regions (Zemach et al., 2013). Details of the RdDM pathways are shown in **Figure 3**. After replication, newly produced DNA will be hemi-methylated at CG and CHG symmetrical sites, but at CHH sites one of the two newly synthesized DNA molecules will not be methylated. Maintenance methylation in the CG context depends on MET1 and VIM1, 2 and 3, and maintenance in the CHG context is catalyzed by CMT3. CHH methylation maintenance depends both on the RdDM pathway and on CMT2 activity. Both CMTs are dependent on histone methylation mediated by KYP and SUVH5 and 6. DNA demethylation can occur passively in a replication dependant way, when the methylation machinery is not or poorly active. 5mC cytosine can be actively removed by DNA glycosylase lyase independently from DNA replication. Newly synthesized DNA strands are highlighted in gray Enzymes names are based on the *Arabidopsis* model. DRM1/2, CMT2/3 (CHROMOMETHYLASE 2/3), MET1 (cytosine-DNA-methyltransferase 1), VIM1–3 (VARIANT IN METHYLATION 1–3), KYP/SUVH4 [KYP/Su-(var)3–9 homolog 4], SUVH5/6 [Su-(var)3–9 homolog 5/6], DRD1 (DEFECTIVE IN RNA-DIRECTED DNA METHYLATION), DDM1 (DECREASE IN DNA METHYLATION), and 24 nt siRNA (24 nucleotide small interfering RNAs).

**FIGURE 3:**
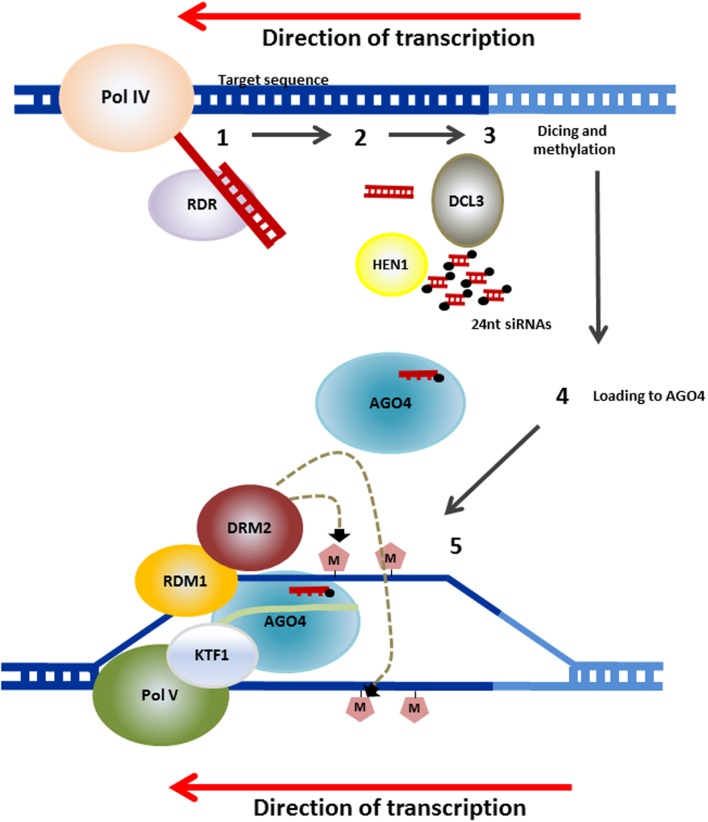
**Mechanism for RdDM.** RNA transcripts are generated from repetitive sequences (transposons and others) by an RNA polymerase known as Pol IV. RNA-DEPENDENT RNA POLYMERASE (RDR) then converts the RNA to double stranded transcripts. These are processed into 24-nucleotide small RNAs (siRNAs) by DICER-LIKE3 (DCL3). These are methylated at their 3′ ends by HUA ENHANCER 1 (HEN1) and the guide strand complementary to the genomic DNA, that will be the target of the RdDM, is incorporated into ARGONAUTE (AGO4). AGO4 is recruited through interactions with Pol V and with KOW DOMAIN-CONTAINING TRANSCRIPTION FACTOR 1 (KTF1). RNA-DIRECTED DNA METHYLATION 1 (RDM1) links AGO4 and DOMAINS REARRANGED METHYLTRANSFERASE 2 (DRM2), which catalyzes *de novo* methylation of DNA (after Matzke and Mosher (2014) and Matzke et al. (2015). Several mechanisms for RdRM have been reported to deviate from this canonical pathway and these are also described in the latter reviews.

Genomic DNA methylation in plants can occur at cytosines in a symmetrical context, CG or CHG, where H is any nucleotide except G or a non-symmetrical context CHH. Cytosine methylation is maintained by a variety of different methyltransferases during DNA replication. Pathways for maintenance of symmetric methylation involve DNA METHYLTRANSFERASE 1 (MET1) which, together with Variant in Methylation proteins 1 and 2 maintains CG methylation (Woo et al., 2008) and CHROMOMETHYLASE (CMT3) which is targeted to specific sequences through its interaction with KRYPTONITE (KYP), SUVH5 and SUVH6, maintains the CHG context (Jackson et al., 2002; Law and Jacobsen, 2010; Du et al., 2014). Asymmetric CHH methylation, which unlike symmetrical methylation, is not found in both daughter DNA molecules, needs an siRNA trigger and requires re-establishment following each cycle of DNA replication and is maintained through persistent *de novo* methylation by the DOMAINS REARRANGED METHYLTRANSFERASE 2 (DRM2) or following a different pathway by CMT2. This requires the nucleosome remodelers DRD1 and DDM1, respectively (**Figure 2**, Kanno et al., 2004; Zemach et al., 2013; Matzke and Mosher, 2014). In the model plant *Arabidopsis*, the mechanism underlying the initiation of methylation marks by DRM2 has been deciphered. This mechanism, known as the RNA-directed DNA methylation (RdDM), is specifically directed at transposons and notably at small and recently acquired transposons in euchromatin. This includes those transposons or repeats in the promoters, introns or coding regions of genes (Matzke and Mosher, 2014). The currently accepted mechanisms of RdDM are summarized in **Figure 3**, and their detailed description is covered in a number of recent publications (Matzke and Mosher, 2014; Bond and Baulcombe, 2015; Matzke et al., 2015).

DNA methylation can also be either lost when active maintenance of DNA methylation is not functional or actively reversed by DNA Glycosylase-Lyases (DNA-GL). DNA-GL, also called DNA demethylases, catalyze the removal of 5mCs which are subsequently replaced by a non-methylated cytosines (**Figure 2**; Gong et al., 2002; Zhu, 2009; Law and Jacobsen, 2010). In *Arabidopsis*, DEMETER, DEMETER-LIKE (DML), and REPRESSOR OF SILENCING 1 (ROS1) recognize and remove methylated cytosines from DNA at specific loci thereby impacting gene expression in developmental processes such as maternal imprinting (Choi et al., 2002; Zhu, 2009; Gehring et al., 2009), male gametophyte development (Schoft et al., 2011), epidermal cell differentiation (Yamamuro et al., 2014) or in response to pathogen attack (Yu et al., 2013). ROS1 activity appears to be regulated through the action of the histone H3 acetyltransferase, INCREASE in DNA METHYLATION 1 (IDM1), an alpha crystalin protein, IDM2, and a Methylcytosine Binding Protein, MBD7 (Qian et al., 2012, 2014; Wang et al., 2015). Recent work has also shown that the final level of DNA methylation is determined by the combined action of both methyltransferases and demethylases in a regulatory loop where *ROS1* gene expression is determined by its methylation level (Lei et al., 2015; Williams et al., 2015).

## Epialleles Can Generate Fleshy Fruit Phenotypic Variations

The potential importance of DNA methylation in sculpting phenotypic variation in tomato was recognized 25 years ago in a study by Messeguer et al. (1991). This study focused on the level, target sites and inheritance of cytosine methylation in nuclear DNA and revealed significant differences in 5mC content between tomato tissues, with highest levels in seeds. Methylation polymorphisms were found between the cultivated tomato (*S. lycopersicum* cv. VF36) and the wild tomato species, *S. pennellii* (LA716) and these polymorphisms were inherited in a normal Mendelian fashion (Messeguer et al., 1991). Hadfield et al. (1993) then reported that a decrease in DNA methylation (DDM) in genes highly expressed in tomato fruits was coincident with the onset of ripening, but the first demonstration that DNA methylation marks could impact ripening was reported in tomato as a result of the cloning of the gene at the *Colourless non-ripening* (*Cnr*) locus (Manning et al., 2006).

The *Cnr* mutant has a non-ripening phenotype where the fruits turn white and then yellow and remain firm (Thompson et al., 1999). The *Cnr* fruits show none of the usual features associated with ripening such as accumulation of carotenoids in the pericarp, softening, or flavor changes (Thompson et al., 1999; Eriksson et al., 2004). The *CNR* gene was cloned using a genetic map-based approach (Manning et al., 2006). Positional cloning delineated a mapping interval of 13 kb containing the *Cnr* locus. This 13 kb region of tomato chromosome 2 harbored three open reading frames and the regulatory region of a fourth gene model. However, there were no sequence differences between mutant and wild-type genomic DNA within the mapping interval. Only one gene model in the 13 kb interval showed strong differential gene expression between mutant and wild type fruits. This gene encoded a SQUAMOSA Promoter Binding Protein (SBP-box/SPL) transcription factor, which are normally associated with control of the expression of *SQUAMOSA* class of MADS-box genes (Manning et al., 2006). Further investigation revealed that part of the regulatory region of this gene was hypermethylated in a 286-bp contiguous region 2.4 kb upstream from the first ATG and this epimark only occurred in lines harboring the *Cnr* mutation (Manning et al., 2006). *Cnr* was a spontaneous mutation and this demonstrates that natural methylation polymorphisms can, under certain circumstances, dramatically affect tomato fruit phenotypes, supporting the potential importance of epigenetic variation in this species as postulated earlier by Messeguer et al. (1991).

A range of natural epialleles affecting fruit phenotypes have now been reported in addition to *Cnr* in tomato and in other plants. A gene encoding a 2-methyl-6-phytylquinol methyltransferase underlying a quantitative trait locus (QTL) for vitamin E from the wild tomato species *S. pennellii* was shown to be associated with differential methylation (Quadrana et al., 2014). Both in apples and pears changes in skin color were associated with hypermethylation of the *MYB10* gene promoter region resulting in repression of this gene expression and the absence of anthocyanin accumulation (Telias et al., 2011; Wang et al., 2013; El-Sharkawy et al., 2015). Very recently, it has been reported that methylation of a CACTA transposon underlies the mantled somaclonal variant of oil palm (*Elaeis guineensis*) fruit (Ong-Abdullah et al., 2015) which is characterized by feminization of flower organs and reduced oil yield.

## How are Epialleles Generated and Maintained?

Epialles as contributors of phenotypic diversity in plants have been produced in the model plant *Arabidopsis* through the generation of EpiRils (Epigenetic Recombinant Inbred lines). Crossing of *ddm1* or *met1* mutants, characterized by hypomethylated genomes, with isogenic wild type parents were used to generate an F_1_ progeny which were genetically identical, but with contrasting sets of DNA methylation marks. The EpiRIL populations were obtained from the F_1_ after seven or eight generations of inbreeding leading to the demonstration that experimentally induced epialleles could stably affect plant traits such as flowering time and plant height, although some reversion was observed (Johannes et al., 2009; Teixeira et al., 2009; Cortijo et al., 2014; Hu et al., 2015; Kooke et al., 2015). However, despite the description of several natural epialleles the mechanisms leading to their generation have remained poorly understood so far. Indeed, genome duplications, which are recognized as important engines of evolution in the Angiosperms (Paterson et al., 2010; Rensing, 2014; Vanneste et al., 2014), might, in addition to the generation of spontaneous mutations, result in transposon movement and in new DNA methylation patterns through the RdDM pathway stimulated by genome shock. It has been estimated that in unstressed *Arabidopsis* the rate of spontaneous gains and losses of DNA methylation is 1000 times higher than the genetic mutation. Whether such genome wide changes in DNA methylation patterns can generate new stable epialleles is an appealing possibility that requires further investigation (Matzke and Mosher, 2014; Matzke et al., 2015). Alternatively, epialleles could be generated following interspecific hybridization as suggested by the analysis of hybrids between *S. lycopersicum* and *S. pennellii*. Results show that there were significant changes in DNA methylation and siRNA populations in the progeny (Shivaprasad et al., 2012). These data provided evidence that phenotypic differences generated following interspecific hybridization in tomato could be due to both epigenetic and genetic variation, and may generate stable epialleles. In several cases epialleles occur in the close vicinity of transposable elements (TEs). For example, the event that initiated the *Cnr* mutation although not yet known, may have arisen because of the proximity of the *CNR* promoter to a Copia-like retrotransposon (Manning et al., 2006) which could direct RdDM to the region of the *Cnr* locus (see work on maize by Gent et al., 2013). Associations between transposon sequences and natural epialleles have also been observed for the *VTE3* gene in tomato (Quadrana et al., 2014), the *FWA* gene in *Arabidopsis* (Lippman et al., 2004), and the *CmWIP1* gene in melon (Martin et al., 2009). All these examples are consistent with the hypothesis that transposons may contribute to the generation of spontaneous epialleles. However, in some cases associations between transposon and natural epialleles were not identified, as for the *CYCLOIDEA* gene in *Linaria vulgarus* (Cubas et al., 1999) and the *MyB A10* gene in pear (Wang et al., 2013) suggesting a diversity of mechanisms being involved in epiallele formation.

The maintenance of many epialleles seems to rely essentially on the normal methylation machinery. Recently Chen et al. (2015) have shown that a CMT that is expressed in developing tomato fruits was up-regulated in the immature fruits of the *Cnr* mutant. Virus induced silencing (VIGS) of this gene in the mutant resulted in increased expression of the *CNR* gene and triggered ripening in the epimutant. VIGS of *SlDRM7*, *SlMET1*, and *SlCMT2* also all had some positive effect on the ripening process in the *Cnr* mutant background. These data indicate that genes involved in DNA maintenance methylation are necessary for the somatic maintenance of this epimutation. A similar observation was made more than a decade ago in *Arabidopsis* by demonstrating that the *clarkent* epiallele of *SUPERMAN* could be reversed by a mutation in the *CMT3* gene (Lindroth et al., 2001). This mutation resulted in a depletion of CHG methylation in *Arabidopsis*, although with no major effect on plant phenotype except for the reversion of the epiallele, demonstrating that the ability to maintain CHG methylation in the superman promoter region was strictly linked to the stability of the epiallele. Mutation of *KYP* a H3 Lys 9 methyltransferase gene had effects similar to mutants in CMT3 with loss of cytosine methylation at CHG sites and reversion of the *clark kent* epiallele (Jackson et al., 2002). This demonstrated the requirement of KYP for CHG maintenance methylation and further illustrates the complex interactions between histone marks and DNA methylation processes (**Figure 3**).

## Fruit Ripening in Tomato Involves Maintenance of DNA Methylation and Requires Active DNA Demethylation

**FIGURE 4:**
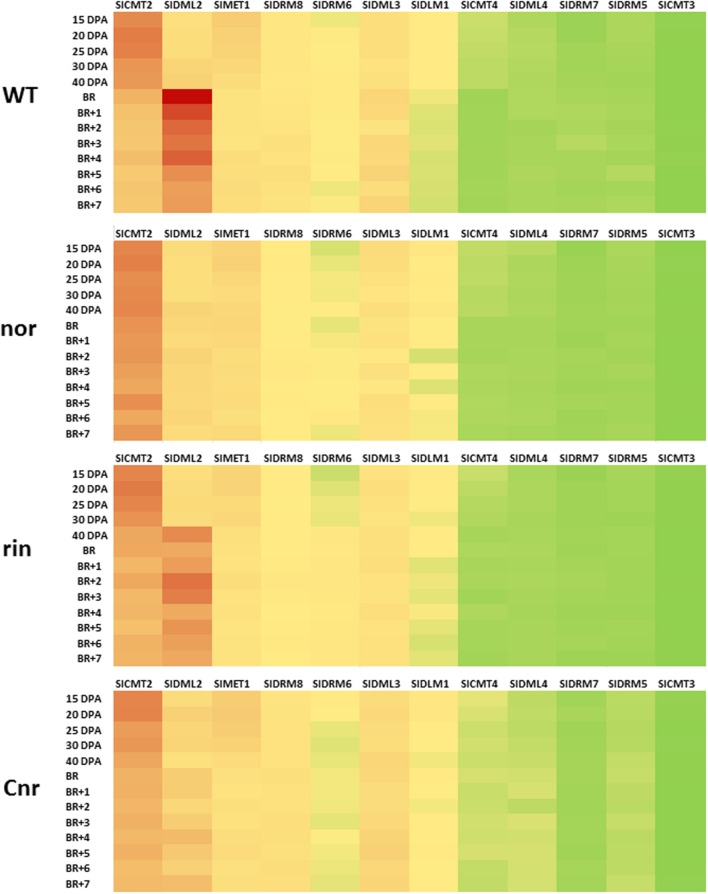
**Expression of methyltransferase and DNA demethylase genes in normal and mutant tomato fruits.** Heat maps from normalized microarray data (see Liu et al., 2015, Supplementary information) showing expression of methyltransferase and DNA demethylase genes in developing and ripening tomato fruits from Ailsa Craig and near isogenic lines containing the *non-ripening* (*nor*), *ripening inhibitor* (*rin*), and *Colourless non-ripening* (*Cnr*) mutations. Fruits were sampled at 15, 20, 25, 30, and 40 days post anthesis (dpa) and also at breaker (Br) and for 7 days post breaker (BR+1 to BR+7). Forty dpa was taken as mature green and BR+7 was the red ripe stage. The values used to construct the maps were means of three biological replicates (individual fruits) at each stage at for each gene. *SlMET1* (*Solanum lycopersicon*, CYTOSINE-DNA-METHYLTRANSFERASE 1), *DML* (*DNA DEMETHYLASE*), *DRM* (*DOMAINS REARRANGED METHYLASE*). On the heat map red is for high levels of gene expression and green for low expression. Yellow represents intermediate values.

In the tomato genome eight 5mC methyltransferases (MTases) and four DMLs genes have been identified (Teyssier et al., 2008; Cao et al., 2014; Chen et al., 2015; Liu et al., 2015). Comparing the protein coding sequences with those of related genes from *Arabidopsis* allows identification of the likely tomato orthologs of genes such as *MET1* and *ROS1* (**Table 2**). For genes involved in maintenance methylation expression analysis based on microarray data (**Figure 4**)^1^ and previous work by Teyssier et al. (2008) indicated that *MET1*, *CMTs*, and several Sl*DRMs* are most active during early fruit development while *SlDRM7* expression peaks during early phases of fruit ripening. The importance of maintenance methylation in determining the onset of ripening was first suggested by the work of Zhong et al. (2013). They reported that treatment of immature tomato fruit with the methyltransferase inhibitor 5-azacytidine could induce premature ripening. During tomato fruit development several rounds of endoreduplication occurs with cells of mature fruits reaching 216 to 512 C depending on the variety (Cheniclet et al., 2005; Teyssier et al., 2008). Hence, in the absence of maintenance methylation the genomes of fruit pericarp cells would gradually become demethylated resulting in the premature induction of the ripening process. The maintenance of DNA methylation in immature fruits is therefore likely to be necessary to block ripening induction before seed maturation.

**Table 2:** Tomato DNA methyltransferases and DNA Glycosylase-Lyase (Demethylase).

Gene accession (Solgene)	Gene id (NCBI)	Proposed name: actual review	*Arabidopsis* ortholog (Gene id)	References
**DNA Methyltransferase**				
Solyc11g030600	543721	SlMET1	AtMET1 (834975)	Teyssier et al., 2008; Cao et al., 2014; Chen et al., 2015
Solyc12g100330	101267211	SlCMT2	AtCMT3 (843313)	Teyssier et al., 2008; Cao et al., 2014; Chen et al., 2015
Solyc01g006100	101265056	SlCMT3	AtCMT3 (843313)	Teyssier et al., 2008; Cao et al., 2014; Chen et al., 2015
Solyc08g005400	101244018	SlCMT4	AtCMT2 (827640)	Teyssier et al., 2008; Cao et al., 2014; Chen et al., 2015
Solyc02g062740	100135704	SlDRM5	AtDRM2 (831315)	Teyssier et al., 2008; Cao et al., 2014; Chen et al., 2015
Solyc10g078190	101266376	SlDRM6	AtDRM1 (831390)	Teyssier et al., 2008; Cao et al., 2014; Chen et al., 2015
Solyc04g005250	101255191	SlDRM7	AtDRM1 (831390)	Teyssier et al., 2008; Cao et al., 2014; Chen et al., 2015
Solyc 05g053260	101267313	SlDRM8	AtDRM3 (820994)	Teyssier et al., 2008; Cao et al., 2014; Chen et al., 2015
Solyc08g067070^∗^		SlDNMT2^∗^	AtDNMT2^∗^ (832623)	Teyssier et al., 2008; Cao et al., 2014; Chen et al., 2015
**DNA Gycosylase Lyases (DNA demethylase)**				
Solyc09g009080	101244311	SlDML1	AtROS1 (818224)	Cao et al., 2014; Liu et al., 2015
Solyc10g083630	101263652	SlDML2	AtROS1 (818224)	Cao et al., 2014; Liu et al., 2015
Solyc11g007580	101252835	SlDML3	AtDEMETER (830335)	Cao et al., 2014; Liu et al., 2015
Solyc03g123440	101251080	SlDML4	ATDML2/AtDML3 (820162)/(829552)	Liu et al., 2015


*^∗^It is unclear whether DNMT2 is an active DNA methyltransferase in plants.*

The importance of DNA demethylation in regulating fruit ripening initially suggested by Hadfield et al. (1993) was highlighted in studies by Teyssier et al. (2008) who showed a 30% decrease of the global DNA methylation levels in tomato pericarp, but not in locular tissues, during tomato fruit maturation. This work suggested tissue specific control of DNA methylation in fruits which is consistent with the tissue dependent differential expression of DNA MTases genes during the development and ripening of fruit tissues (Teyssier et al., 2008). However, the DDM observed in fruit pericarp occurred when cell division and endoreduplication is limited, making unlikely a replication dependent passive loss of DNA methylation (Teyssier et al., 2008, **Figure 4**). This was consistent with locus-specific loss of DNA methylation in ripening-related genes reported by Hadfield et al. (1993) who showed a decrease in methylation at the *POLYGALACTURONASE* (PG) and *CELLULASE* gene promoters at the onset of tomato ripening and more recently similar changes in the *CNR* promoter in the cultivar Liberto (Manning et al., 2006).

A breakthrough study providing new insights into the importance of DNA demethylation in ripening was reported by Zhong et al. (2013). In a genome wide analysis of DNA methylation in tomato they found dynamic changes in 5mC distribution during fruit development and revealed a loss of 5mC in the promoters of more than 200 ripening-related genes, a list of which can be found in Zhong et al. (2013; Supplementary Tables S10 and S12). These included genes encoding proteins involved in carotenoid accumulation (PHYTOENE SYNTHASE: PSY1; 15-CIS-ZETA-CAROTENE ISOMERASE), in ethylene synthesis (ACO1, ACS2) and reception (NR, ETR4), in fruit softening (PG; PECTIN METHYLESTERASE: PMEU1), and several transcription factors of various classes (MADS-box, WRKY, or NAC), among which those controlling ripening induction such as RIPENING INHIBITOR (RIN), NON-RIPENING (NOR), COLORLESS NON-RIPENING (CNR), and TAGL1. The differentially methylated regions in these genes were typically adjacent to binding sites for RIN (Zhong et al., 2013), a MADS-box transcription factor that acts as a master regulator of ripening in tomato (Vrebalov et al., 2002). In addition to providing compelling evidence that ripening is governed by epigenetic in addition to genetic and other components, these data indicated that demethylation does not occur in a random way, but is rather targeted at specific sites, again consistent with active DNA demethylation being intimately involved in the ripening process.

Liu et al. (2015) have now been able to demonstrate that active DNA demethylation is the mechanism responsible for the loss in 5mC at the onset of ripening. They showed that among the four potential DNA demethylases found in the tomato genome, there was one gene, *SlDML2*, which was strongly induced at the onset of ripening concomitantly with the DDM (Teyssier et al., 2008; Zhong et al., 2013). RNAi or VIGS mediated *SlDML2* silencing resulted in extremely delayed ripening and ripening defects associated with repression of essential ripening induced transcription factors and of *PSY1*, which controls carotenoid accumulation during ripening. Silencing of these genes was correlated to the hypermethylation of their promoter regions in contrast to their demethylation in WT fruits. This causal relationship between active demethylation and induction of fruit ripening demonstrated that there is an epigenetic layer of control for fruit ripening, at least in tomato.

**FIGURE 5:**
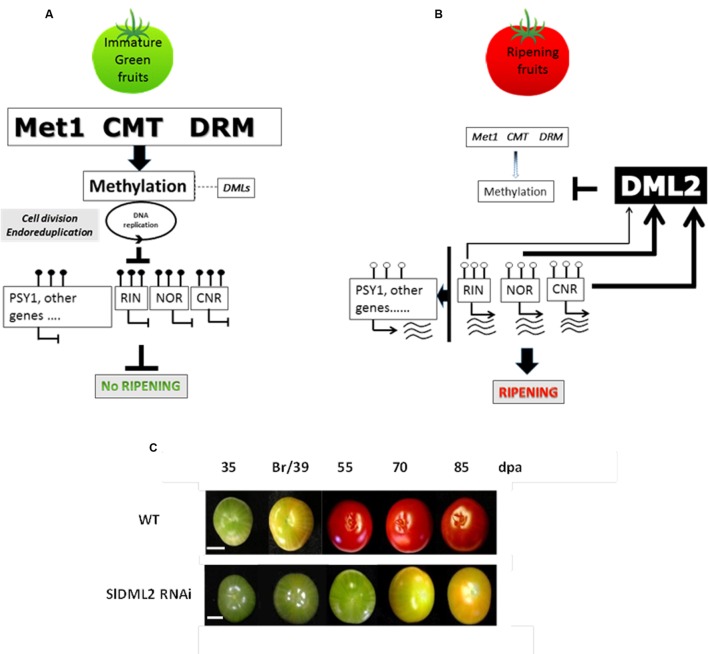
**Model of molecular framework linking the induction of ripening with the action of methyltransferases, DNA demethylases and DNA methylation.** **(A)** The *RIN*, *NOR*, and *CNR* genes encode master regulators of ripening. Their expression in immature tomato fruit is inhibited along with that of other ripening genes including *PSY1* by 5mC marks in their regulatory regions. Potential additional targets are listed in the Supplementary Table S10 of Zhong et al. (2013) and include genes involved in ethylene biosynthesis and perception, fruit softening as well as many transcription factors of various classes. These are maintained in immature green fruit. **(B)** However, at the onset of ripening these epi-marks are removed by DNA demethylases. Expression of the *NOR*, *RIN*, and *CNR* genes then occurs and the ripening process is initiated. SlMET1 (cytosine-DNA-methyltransferase 1), CMT (CHROMOMETHYLASE), DRM. **(C)** Example of fruits from transgenic RNAi plants affected for SlDML2 gene expression (adapted from Liu et al., 2015).

In addition, *SlDML2* was shown to be down regulated in the *Cnr* and *nor* backgrounds, and to a lower extent in a *rin* background, suggesting a regulatory loop between transcription factors controlling fruit ripening and DNA demethylation (**Figure 5**). Liu et al. (2015) also reported that the hypermethylation of the genomic DNA of *Cnr* and *rin* fruit occurred to a level and intensity that was correlated with the repression level of *SlDML2* in the corresponding mutant fruits. The demonstration that *SlDML2* is also repressed in the *nor* mutant background indicates that genomic DNA in this mutant may be hypermethylated to a similar extent as in *Cnr*. It is possible that the ripening defects in *rin*, *nor*, and *Cnr* may, at least in part, be due to limited demethylation in addition to, and as a result of, the absence of these transcription factors. Whether *SlCMT2* which is upregulated in *Cnr* during fruit ripening (**Figure 4**), also contributes to the hypermethylated phenotype observed in these fruits is so far unclear, as the increase in 5mC levels are not limited to the CHG context normally mediated by CMT enzymes, but occurs in all sequence contexts (Zhong et al., 2013).

## Conclusion

Recent work on various plants including *Arabidopsis* (Zhang et al., 2006; Zilberman et al., 2007; Cokus et al., 2008), rice (Li et al., 2012), maize (Gent et al., 2013), and tomato (Zhong et al., 2013) has demonstrated that remodeling of epigenomes occurs at various stages during plant development. Indeed, *Arabidopsis* plants with altered control of histones PTMs or hypomethylated genomes present numerous phenotypes consistent with epigenome homeostasis being critically important for proper plant development (Finnegan et al., 1996), but also adaptation to environmental changes (Baulcombe and Dean, 2014). Considering the plethora of enzymes involved in the control of histone PTMs (Kouzarides, 2007; Lauria and Rossi, 2011) and their complex expression patterns in fleshy fruits (Janssen et al., 2008; Aquea et al., 2010, 2011; Almada et al., 2011; Cigliano et al., 2013; Zhao et al., 2014; Xu et al., 2015a), it is very likely that they will be involved in several aspect of this development process. Among them, the H3K27me3mark, established by the Polycomb group proteins, appears to be important at early stages of tomato fruit development (How Kit et al., 2010; Liu et al., 2012; Boureau et al., 2016). Yet, there is still much to do to get a clear understanding of the precise function of histone modifications in fruits as most studies performed so far are correlative, and functional analysis of the histone modifiers is now necessary. It is also unclear to which extent variations in histone PTMs will be stably inherited and impact fruit phenotypes across generations. Alternatively, it is also plausible that genetic diversity of histone modifiers (diversification of gene families) as well as changes in their expression pattern could contribute to shape epigenetic driven phenotypic changes within or between species.

The understanding of the functions of DNA methylation in fleshy fruits is by far more advanced than that relating to histone PTMs, at least in the tomato plant. The results discussed in this review clearly show that fruit ripening is under strict epigenetic control mediated by changes in DNA methylation levels and distribution, in addition to genetic and hormonal controls (for review Gapper et al., 2013). The current model of ripening proposes that active demethylation is necessary to trigger fruit ripening (**Figure 5**, Liu et al., 2015), and this process should target several hundred of genes as shown by the methylome analysis in ripening fruits (Zhong et al., 2013). Changes in DNA methylation patterns might therefore play a more important role in the control of gene expression during plant developmental processes than anticipated from previous studies mainly based on the *Arabidopsis* model (Eichten et al., 2014). Indeed, when considering DNA methylation *Arabidopsis* may be an “*epigenetic exception*” with only 5% of methylated cytosine in the genome (Lister et al., 2008) and very few TEs, limiting the likelihood for DNA methylation control of gene expression. This contrasts with TE and DNA methylation-rich crops that contain more than 20% of methylated cytosines in their genomes (Teyssier et al., 2008; Li et al., 2012; Gent et al., 2013) and high transposon contents (Tenaillon et al., 2010; Lee and Kim, 2014). In addition the distribution of DNA methylation also differs between *Arabidopsis* and other plants including tomato or maize where a substantial proportion of methylation is in the CHH context (Gent et al., 2013; Zhong et al., 2013). Thus DNA methylation may play more important role in plant species with more ‘complex’ genomes as illustrated by its central function in tomato fruit ripening.

In the context of tomato fruits, it is possible to speculate that the regulation of ripening mediated by the DNA methylation/demethylation balance has evolved as a ‘double-lock’ mechanism, along with changes in gene expression as a result of developmental cues, to prevent premature dispersal of seeds prior to their full maturation. It remains now to be determined whether the epigenetic control of ripening has emerged similarly in other types fleshy fruits or is limited to the tomato and related wild species.

In relation to crop improvement and breeding strategies, epi-marks on gene promoter regions could be used for ‘fine tuning’ of gene expression. Examples published for tomato include the biosynthesis of vitamin E and gene expression at the *Cnr* locus. VTE3 gene expression in Andean landraces of tomato (*S. lycopersicum*) and commercial cultivars is related to the extent of methylation in the VTE3 promoter region (Quadrana et al., 2014) and differences in the extent of methylation in the *CNR* promoter are apparent in normally ripening fruits of the cultivars Liberto and Ailsa Craig. Higher levels of expression of *CNR* in Ailsa Craig, in comparison to Liberto, are associated with reduced DNA methylation in a region of the gene upstream of the first ATG (Manning et al., 2006). A comprehensive analysis of the distribution of epi-marks and DNA methylation in tomato and other fruit crops in relation with gene expression profiles and fruit quality traits would likely identify epialleles that could be used as important new targets for plant breeding.

## Author Contributions

CH provided experimental data and helped write the manuscript. ET helped write the article. PG and GS conceived the review, provided data and wrote the manuscript.

## Conflict of Interest Statement

The authors declare that the research was conducted in the absence of any commercial or financial relationships that could be construed as a potential conflict of interest.

## Footnotes

1 ftp://ftp.solgenomics.net/microarray/
